# Supplementary material for: Effect of Irrigation and Fertilizer Management on Rice Yield and Nitrogen Loss: A Meta-Analysis
Source: Plants (Basel). 2022 Jun 26;11(13):1690. doi: 10.3390/plants11131690 (PMC9268946; doi:10.3390/plants11131690)
Supplement: Supplementary file 1 [file plants-11-01690-s001.zip › plants-1780269-supplementary.pdf]

Table S1.The effect of irrigation schedules on yield (P=0.00000)

W	study	E	CIlower	CIupper	Z	ZC1	ZC2	Emin	Emax	SD
W2	39	0.007	-0.015	0.029	0.692	-1.528	2.973	-0.132	0.329	0.023
W1	89	0.025	0.013	0.038	2.573	1.298	3.863	-0.207	0.456	0.051
W9	14	0.025	-0.010	0.060	2.491	-1.035	6.141	-0.069	0.092	0.034
W4	8	-0.239	-0.312	-0.166	-21.251	-26.765	-15.321	-0.367	0.004	0.067
W7	3	0.043	-0.191	0.278	4.415	-17.420	32.022	0.033	0.056	0.000
W5	9	-0.080	-0.220	0.061	-7.670	-19.756	6.237	-0.170	-0.016	0.000

Table S2. The effect of nitrogen application on yield (P=0.00011).

N	study	E	CIlower	CIupper	Z	ZC1	ZC2	Emin	Emax	SD
N5	29	0.4516	0.396	0.508	57.082	48.542	66.113	0.170	0.741	0.077
N2	23	0.1958	0.132	0.260	21.628	14.065	29.693	0.067	0.449	0.047
N3	47	0.2621	0.221	0.303	29.966	24.732	35.419	0.104	0.615	0.099
N4	46	0.3632	0.321	0.406	43.792	37.823	50.020	0.006	0.700	0.198
N8	9	0.3855	0.278	0.493	47.035	31.996	63.788	0.190	0.491	0.093
N7	4	0.2975	-0.079	0.674	34.649	-7.550	96.109	0.203	0.413	0.000
N6	3	0.2974	-0.090	0.685	34.635	-8.625	98.377	0.259	0.324	0.000

Table S3. The effect of water-nitrogen coupling on yield (P=0.00987).

combination	study	E	CIlower	CIupper	Z	ZC1	ZC2	Emin	Emax	SD
W1N5	4	0.437	0.224	0.650	54.806	25.145	91.497	0.217	0.601	0.087
W1N3	16	0.246	0.178	0.315	27.941	19.518	36.957	0.131	0.429	0.078
W1N4	19	0.393	0.330	0.456	48.083	39.055	57.696	0.071	0.621	0.175
W2N2	3	0.164	-0.158	0.486	17.857	-14.606	62.645	0.124	0.228	0.023
W2N3	5	0.318	0.155	0.481	37.396	16.719	61.737	0.187	0.565	0.062
W3N2	2	0.141	-0.980	1.263	15.177	-62.469	253.460	0.160	0.385	0.068
W3N4	2	0.219	-0.903	1.340	24.458	-59.461	282.057	-0.001	0.346	0.112
W1N2	4	0.241	0.036	0.446	27.265	3.645	56.268	0.217	0.601	0.087
W4N4	5	0.109	-0.073	0.292	11.561	-7.068	33.924	0.131	0.429	0.078
W5N2	2	0.045	-1.980	2.071	4.645	-86.199	693.434	0.071	0.621	0.175
W5N4	2	0.208	-2.008	2.424	23.109	-86.580	1029.319	0.124	0.228	0.023
W5N5	2	0.262	-1.841	2.366	29.992	-84.139	965.362	0.187	0.565	0.062
W5N7	2	0.199	-1.868	2.267	22.055	-84.557	864.558	0.160	0.385	0.068

Table S4. The effect of irrigation schedules on nitrogen use efficiency (P= 0.01682).

W	study	E	CIlower	CIupper	Emin	Emax	SD
W1	27	0.474	0.078	0.870	-2.400	6.586	0.747
W2	13	1.064	0.466	1.663	0.404	2.545	0.000
W4	4	-1.637	-3.322	0.048	-3.020	-1.164	0.000

Table S5. The effect of nitrogen application on nitrogen use efficiency (P=0.03588).

N	study	E	CIlower	CIupper	Emin	Emax	SD
N4	51	11.923	10.467	13.380	2.715	240.982	4.208
N5	18	13.494	10.727	16.260	9.229	239.474	3.437
N2	6	8.776	4.220	13.333	5.325	12.298	1.268
N3	22	11.758	9.523	13.993	6.311	49.898	2.333
N6	4	12.257	4.298	20.216	11.300	14.624	0.000
N9	4	15.353	6.235	24.471	11.303	18.583	0.000

Table S6. The effect of water-nitrogen coupling on nitrogen use efficiency (P=0.01631).

combination	study	E	CIlower	CIupper	Emin	Emax	SD
W1N5	3	16.259	0.978	31.541	11.314	22.858	4.473
W1N4	13	9.595	6.607	12.584	2.715	240.982	4.614
W2N4	7	15.750	10.105	21.394	11.306	37.924	3.422
W4N4	4	11.312	3.483	19.140	11.300	11.326	0.000
W1N3	8	13.135	8.566	17.704	11.314	32.527	1.368
W2N9	4	15.383	6.093	24.673	11.303	18.583	0.000

Table S7. The effect of irrigation schedules on NO_2 (P=0.00004).

W	study	E	CIlower	CIupper	Z	ZC1	ZC2	Emin	Emax	SD
W1	35	0.517	0.426	0.609	67.766	53.066	83.878	0.000	1.658	0.299
W2	20	0.060	-0.065	0.184	6.162	-6.246	20.214	-0.590	0.445	0.214
W9	6	0.357	0.085	0.629	42.846	8.828	87.480	0.235	0.521	0.066
W6	5	0.121	-0.213	0.454	12.806	-19.168	57.444	0.098	0.140	0.000

Table S8. The effect of nitrogen application on NO_2 (P=0.00538).

N	study	E	CIlower	CIupper	Z	ZC1	ZC2	Emin	Emax	SD
N4	17	0.657	0.514	0.800	92.861	67.197	122.443	0.162	1.671	0.297
N2	15	0.366	0.212	0.519	44.123	23.627	68.035	0.060	0.661	0.187
N3	31	0.581	0.478	0.683	78.711	61.317	97.961	-0.329	1.484	0.281

Table S9. The effect of water-nitrogen coupling on NO_2 (P=0.00000).

combination	study	E	CIlower	CIupper	Z	ZC1	ZC2	Emin	Emax	SD
W2N2	5	0.566	0.330	0.802	76.033	39.041	122.888	0.486	0.731	0.059
W2N3	9	0.824	0.677	0.970	127.892	96.856	163.821	0.661	1.219	0.144
W1N4	7	0.919	0.743	1.095	150.703	110.223	199.008	0.301	1.990	0.135
W1N3	7	1.069	0.891	1.246	191.188	143.830	247.710	0.364	1.851	0.296
W1N2	3	0.677	0.214	1.140	96.856	23.899	212.771	0.094	0.966	0.399

Table S10. The effect of irrigation schedules on nitrogen runoff (P=0.00138).

W	study	E	CIlower	CIupper	Z	ZC1	ZC2	Emin	Emax	SD
W2	6	-0.215	-0.595	0.166	-19.306	-44.855	18.081	-0.326	-0.057	0.000
W1	21	-0.397	-0.544	-0.250	-32.787	-41.969	-22.151	-0.994	0.125	0.366
W5	3	-1.085	-1.887	-0.283	-66.213	-84.846	-24.678	-1.107	-1.050	0.000
W7	3	0.420	-0.382	1.221	52.135	-31.757	239.159	0.394	0.445	0.000

Table S11. The effect of nitrogen application on nitrogen runoff (P=0.52096).

N	study	E	CIlower	CIupper	Z	ZC1	ZC2	Emin	Emax	SD
N4	15	0.696	0.347	1.045	100.511	41.453	184.226	0.121	1.850	0.524
N7	5	0.818	-0.030	1.666	126.551	-2.955	428.832	0.617	1.314	0.122
N3	10	0.960	0.524	1.397	161.274	68.911	304.184	0.068	1.856	0.706
N5	14	1.372	1.020	1.723	294.165	177.319	460.299	0.337	2.271	0.730
N2	3	0.958	-0.557	2.472	160.544	-42.725	1085.085	0.761	1.078	0.151
N6	5	0.760	-0.085	1.604	113.785	-8.140	397.487	-0.273	1.792	0.516

Table S12. The effect of water-nitrogen coupling on nitrogen runoff (P=0.53041).

combination	study	E	CIlower	CIupper	Z	ZC1	ZC2	Emin	Emax	SD
W1N3	2.000	1.599	-4.670	7.868	394.957	-99.062	261185.821	0.121	1.850	0.524
W1N5	3.000	1.340	-0.394	3.073	281.752	-32.544	2060.663	0.617	1.314	0.122

Table S13. The effect of irrigation schedules on nitrogen leaching (P=0.00000).

W	study	E	CIlower	CIupper	Z	ZC1	ZC2	Emin	Emax	SD
W1	15	-0.154	-0.258	-0.049	-14.239	-22.748	-4.791	-0.550	0.126	0.280
W2	24	-0.676	-0.752	-0.599	-49.125	-52.867	-45.086	-1.140	-0.185	0.169
W7	3	-0.319	-0.791	0.153	-27.327	-54.670	16.498	-0.386	-0.258	0.000
W8	5	-0.432	-0.668	-0.196	-35.053	-48.701	-17.774	-0.527	-0.388	0.000

Table S14. The effect of nitrogen application on nitrogen leaching (P=0.44466).

N	study	E	CIlower	CIupper	Z	ZC1	ZC2	Emin	Emax	SD
N3	9	0.351	-0.008	0.710	42.077	-0.757	103.379	0.012	0.823	0.214
N4	9	0.731	0.372	1.089	107.633	45.063	197.190	0.222	1.216	0.297
N5	15	0.677	0.422	0.932	96.796	52.501	153.958	-0.272	1.337	0.506
N7	3	0.519	-0.612	1.650	67.984	-45.779	420.490	0.019	1.420	0.648

Table S15. The effect of water-nitrogen coupling on nitrogen leaching (P=0.24925).

combination	study	E	CIlower	CIupper	Z	ZC1	ZC2	Emin	Emax	SD
W1N3	2	0.067	-8.969	9.102	6.908	-99.987	897222.121	0.000	0.134	0.047
W1N5	3	1.487	-1.011	3.985	342.380	-63.618	5279.605	0.406	2.825	1.227

Table S16. The effect of irrigation schedules on nitrogen NH_3 (P=0.02987).

W	study	E	CIlower	CIupper	Z	ZC1	ZC2	Emin	Emax	SD
W1	13	-0.004	-0.097	0.088	-0.439	-9.244	9.210	-0.257	0.323	0.187
W2	17	-0.235	-0.312	-0.159	-20.975	-26.831	-14.658	-0.474	0.159	0.084

Table S17. The effect of nitrogen application on nitrogen NH_3 (P=0.00000).

N	study	E	CIlower	CIupper	Z	ZC1	ZC2	Emin	Emax	SD
N4	27	1.785	1.518	2.051	495.660	356.355	677.567	0.791	3.419	0.676
N3.	17	2.305	1.957	2.654	902.719	607.665	1320.935	1.038	3.526	0.749
N5	21	2.839	2.531	3.146	1609.011	1156.355	2224.756	1.421	4.509	0.624
N6	9	2.677	2.150	3.205	1354.577	758.228	2365.303	1.927	3.762	0.557
N7	10	3.350	2.872	3.828	2748.849	1666.526	4494.753	1.999	5.202	0.636

Table S18. The effect of water-nitrogen coupling on nitrogen NH_3 (P= 0.09947).

**combination**	**study**	**E**	**CIlower**	**CIupper**	**Z**	**ZC1**	**ZC2**	0.815	3.372	1.386
W1N3	3	2.406	-0.045	4.857	1009.062	-4.391	12765.057	1.056	1.236	0.098
W1N4	2	1.146	-7.719	10.010	214.464	-99.956	2225573.637	3.584	3.800	0.130
W1N5	2	3.692	-5.173	12.557	3912.100	-99.433	28396221.951	0.815	3.372	1.386
